# First incidence of extrarenal wilms tumor within the spinal canal in the adult population: a novel case report and literature review

**DOI:** 10.1186/s12894-024-01508-6

**Published:** 2024-06-10

**Authors:** Babak Alijani, Elahe Abbaspour, Sahand Karimzadhagh, Zoheir Reihanian, Mohammad Haghani Dogahe, Maryam Jafari, Seifollah Jafari, Nooshin Zaresharifi

**Affiliations:** 1 Department of Neurosurgery, Poursina Hospital, Guilan University of Medical Sciences, Rasht, Iran; 2 Neuroscience Research Center, Guilan University of Medical Sciences, Rasht, Iran; 3 Department of Radiology, Poursina Hospital, Guilan University of Medical Science, Rasht, Iran; 4 Clinical Research Development Unit of Poursina Hospital, Guilan University of Medical Sciences, Rasht, Iran; 5 Student Research Committee, School of Medicine, Anzali International Campus, Guilan University of Medical Sciences, Rasht, Iran; 6 Neuroscience Research Center, Guilan University of Medical Sciences, Rasht, 41937-13194 Iran

**Keywords:** Wilms tumor, Nephroblastoma, Extrarenal Wilms tumor, Extra-renal nephroblastoma, Spinal dysraphism, Spinal tumors

## Abstract

**Background:**

Wilms tumor (WT), also known as nephroblastoma, is rare in adults, accounting for merely 3% of all nephroblastomas or 0.2 cases per million individuals. Extrarenal Wilms tumor (ERWT) emerges outside the renal boundaries and comprises 0.5 to 1% of all WT cases, with even rarer incidences in adults. Oncogenic mutations associated with ectopic nephrogenic rests (NR) may contribute to ERWT development. Diagnosis involves surgical resection and pathology examination. Due to scarce cases, adults often rely on pediatric guidelines. We thoroughly searched PubMed, Scopus, and Web of Science databases to establish our case’s uniqueness. To the best of our knowledge, this is the first documented incidence of extrarenal Wilms tumor within the spinal canal in the adult population.

**Case presentation:**

A 22-year-old woman with a history of congenital lipo-myelomeningocele surgery as an infant presented with a 6-month history of back pain. This pain gradually resulted in limb weakness, paraparesis, and loss of bladder and bowel control. An MRI showed a 6 × 5 × 3 cm spinal canal mass at the L4-S1 level. Consequently, a laminectomy was performed at the L4-L5 level to remove the intramedullary tumor. Post-surgery histopathology and immunohistochemistry confirmed the tumor as ERWT with favorable histology without any teratomatous component.

**Conclusion:**

This report underscores the rarity of extrarenal Wilms tumor (ERWT) in adults, challenging conventional assumptions about its typical age of occurrence. It emphasizes the importance of clinical awareness regarding such uncommon cases. Moreover, the co-occurrence of spinal ERWTs and a history of spinal anomalies warrants further investigation.

## Introduction

Wilms tumor (WT) or nephroblastoma occurrence in adults is uncommon; only 3% of all nephroblastomas are reported in adults, equating to 0.2 cases per million individuals [1]. Extrarenal Wilms’ tumor (ERWT) is an exceptionally scarce variant of Wilms’ tumor, presenting outside the renal boundaries. While more prevalent in children, ERWT in adults signifies an even rarer incidence [2]. ERWT occurrences within the spinal cord are exceptionally rare, with only a handful of documented cases reported, primarily in pediatric patients. To our knowledge, no prior instances of an intramedullary ERWT within the adult population have been reported. Remarkably, our case represents the first occurrence of such an entity, found in a 22-year-old female patient.

The precise origins of ERWT’s development still need to be fully understood, prompting the exploration of numerous hypotheses. The most widely embraced hypothesis proposes that ectopic nephrogenic rests (NR), aggregates of embryonal renal tissue persisting beyond the 35th week of development, can be attributed to the development of ERWTs through oncogenic mutation. Moreover, existing studies suggest that incomplete neural tube closure in the fourth week of embryonic life can hinder the migration of developing metanephric blastema, coinciding with the onset of metanephric kidney formation [3, 4]. Due to the limited reported cases, finding an optimal treatment for this tumor and predicting the prognosis is challenging. While childhood Wilms tumors have a high cure rate, outcomes in adults are less successful [5]. Here, we present the first incidence of a unique case involving a 22-year-old female with a history of lipo-myelomeningocele surgery during the neonatal period who presented with ERWT in the spine after 22 years.

## Case Presentation

A 22-year-old female patient was admitted to the hospital with a history of back pain and progressive lower limb weakness, persisting for six months. Remarkably, she did not report any prior history of mobility or voiding difficulties. However, her lower limb weakness worsened progressively, leading to paraparesis and loss of bladder and bowel control. Notably, the patient had a medical history of congenital lipo-myelomeningocele, which had been surgically repaired in infancy. Nevertheless, she did not report any post-operative incontinence or neurological issues during childhood. On physical examination, her vital signs were stable; however, a neurological assessment revealed flaccid paralysis in both lower limbs, absent deep tendon reflexes, and sensory deficits in the lower limbs.

**Fig. 1:**
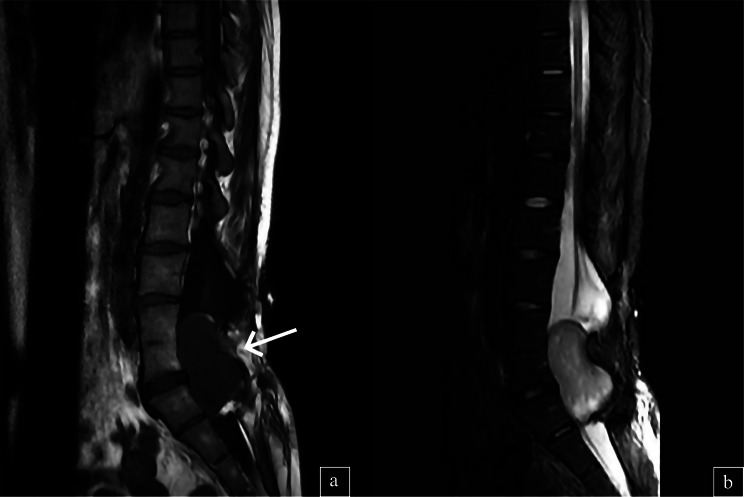
(**a**) T1-weighted lumbar MRI image showed a well-defined hypointense mass (arrow) within the spinal canal at the L4-S1 level. (**b**) In the sagittal T2-weighted MRI image, the lesion measuring 6 × 5 × 3 cm appeared hyperintense

Radiological assessment, including spinal magnetic resonance imaging (MRI), identified a well-defined mass measuring 6 × 5 × 3 cm located within the spinal canal at the L4-S1 level. Considering the patient’s history of myelomeningocele, the differential diagnosis included primary spinal cord tumors and secondary lesions. **(**Fig. 1**)**

Subsequently, the patient underwent a laminectomy at the L4-L5 level to surgically excise the intramedullary tumor, with the primary goals being the relief of spinal cord compression and the preservation of neurological function. The postoperative period proceeded without any notable complications.

**Fig. 2:**
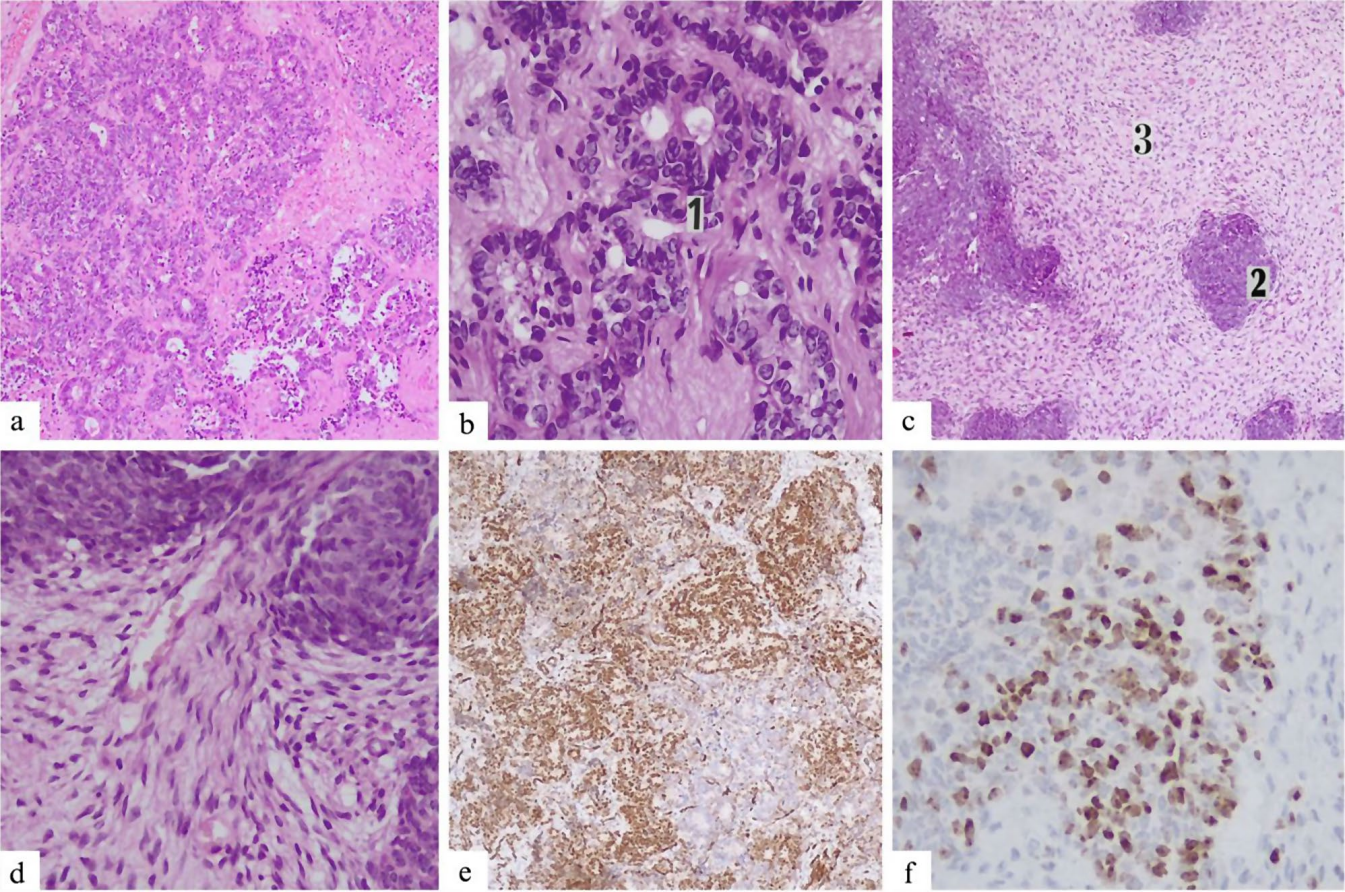
(**a**) Microscopic examination showing triphasic histology (H&E, ×100). (**b**) 1. Epithelial component with glomeruloid and tubular structures (H&E, ×400) (**c**) 2. Blastema component in the stromal background featuring primitive round/oval blastomatous cells and 3. spindle-shaped stromal cells, without any teratomatous elements (H&E, ×100). (**d**) Within the same specimen at a higher magnification (H&E, ×400) (**e**, **f**) WT-1 positive reaction of the blastema component, immunohistochemical staining (×100 & ×400)

The postoperative histopathologic examination identified an extrarenal Wilms tumor displaying a favorable histological pattern. Distinctive histological features included a triphasic pattern characterized by (1) blastemal elements consisting of primitive cells with sporadic mitotic figures, (2) stromal components comprising spindle-shaped cells, and (3) small areas of epithelial tissue displaying glomeruloid and tubular structures. No signs of anaplasia or teratoma were observed. The immunohistochemistry study (IHC) confirmed the histologic diagnosis with a strong WT1 expression in the blastemal region (Fig. 2).

Later, additional imaging studies, including abdominal ultrasonography (USG), were conducted, revealing kidneys of normal size with a healthy cortex and sinus. However, both kidneys exhibited increased parenchymal thickness. Furthermore, a 4 mm stone was detected in the mid-pole of the right kidney, with no signs of hydronephrosis. Aside from these findings, no other significant observations were made.

Computed tomography (CT) scans of the chest, abdomen, and pelvis were also performed to exclude the possibility of a primary renal tumor or any tumor originating outside the kidneys. The results were normal and without any abnormalities.

Regular follow-up assessments, comprising clinical examinations and imaging studies, were scheduled to monitor for potential recurrence. Post-operative follow-up revealed significant improvement in lower limb strength, reaching 3/5 both proximally and distally. However, incontinence remained unresolved. The patient was referred for further evaluation to customize the chemotherapy regimen to the patient’s specific needs, aiming to minimize the risk of recurrence. Unfortunately, during subsequent follow-ups, it was discovered that due to low socioeconomic conditions, the patient did not adhere to her treatment plan.

## Discussion

We report a case of a 22-year-old female with a history of repaired lipo-myelomeningocele presented with progressive back pain and lower limb weakness. The subsequent MRI revealed an intramedullary tumor at L4-S1. Following laminectomy, tumor excision, and histopathological examination, a diagnosis of ERWT was confirmed. Postoperatively, despite persisting incontinence, lower limb strength improved significantly. Despite efforts to customize treatment to the patient’s needs, adherence to the recommended plan during follow-up was hindered by low socioeconomic status.

In approximately 90% of ERWT cases, the diagnosis is established before the age of 7, with most cases occurring before 5 [6]. Therefore, to explore the rarity of our case, we conducted an extensive search across PubMed, Scopus, and Web of Science databases using specific keywords, including “Extrarenal,” “ERWT,” “Wilms,” “Nephroblastoma,” “spine,” “spinal,” “intraspinal,” and “intramedullary.” Our most recent search update on September 10, 2023, identified a total of 272 articles since inception. We narrowed our focus to case series and case reports after excluding duplicates (82 articles), non-English articles, reviews, and cases involving non-human subjects mentioned solely in the article titles and abstracts. Thoroughly examining the full text of the remaining 45 studies, we identified 10 cases of spinal ERWTs. Based on the information available in the literature, our case presents a novel occurrence of extrarenal Wilms tumor within the spinal canal in an adult. Table 1 provides an overview of reported cases of ERWT within the spinal canal as documented in the existing literature.

**Table 1:** Overview of ERWT cases reported within the spin in the literature

Author	Patient	Age	Location	Anomaly/Birth stigmata	Outcome and follow-up
Present Case	Female	22-year-old	L4-S1	Spinal dysraphism	Unresolved incontinency
Karim et al. [27], 2022	Male	4-year-old	T12-S3	Spinal dysraphism (posterior spina bifida)	The patient died in the intensive care unit two years later.
Tokuç et al. [28], 2022	Female	3-year-old	L2-S2	Dermal sinus and tethered cord diagnoses	weakness in her right lower extremity (muscle strength 4/5))
Igbaseimokumo et al. [16], 2017	Female	Newborn	L5	Occult dysraphism/ Dorsal Lipoma with hypertrichosis	No recurrence at 1 years
Wu et al. [4], 2014	Male	9-month-old	T7-L1	Vertebral malformation, meningomyelocele, diastematomyelia and tethered cord syndrome	No recurrence at 6 months
Sharma et al. [29], 2009	Male	20-day-old	L4-L5	Lumbar spina bifida/ tethered cord	No recurrence at 9 months
Deshpande et al. [30], 2002	Male	1-year-old	L2-L4	No previous stigmata	Receiving treatment at the time of the report 3 months later
Govender et al. [31], 2000	Female	4-year-old	T10	Spina bifida occulta	Palliative care only
Fahner et al. [32], 1993	Female	2.5-year-old	L5	Lipoma with skin dimple	No recurrence at 2 years
Mirkin et al. [33], 1990	Female	4- year-old	T12 - L4	Diastematomyelia /Spina Bifida with hypertrichosis	Metastasis to cerebellum 1 year later
Fernbach et al. [34], 1984	Female	2- year-old	L1	Diastematomyelia/ hypertrichosis	No recurrence at 1 year

**Fig. 3:**
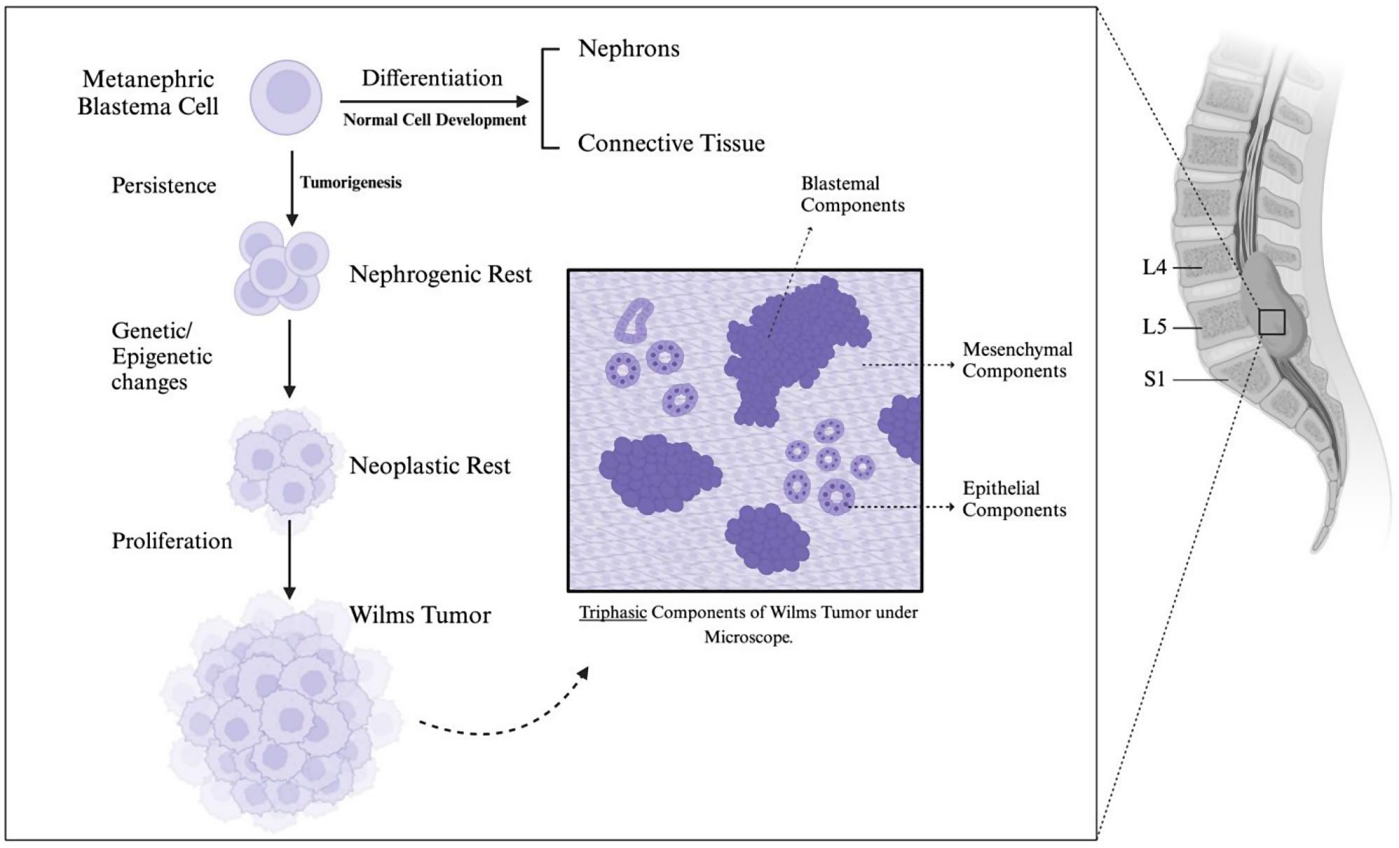
Schematic Presentation of normal kidney development and tumorigenesis of Wilms tumor. The classical histopathological feature is the triphasic pattern comprising blastemal, epithelial, and stromal components

ERWT was initially documented by Moyson et al. in 1961, representing approximately 0.5 to 1% of all Wilms tumor diagnoses [7]. Adult WTs are rare and share histological characteristics with their pediatric counterparts, leading to similar treatment approaches for both age groups. However, a notable disparity exists in their behavior, with adult WTs often displaying more aggressive behavior and limited responses to treatment [8]. Most ERWTs are diagnosed following surgical resection and pathological assessment. Since there are no distinguishing histological features between adult and pediatric Wilms’ tumors, three diagnostic criteria have been established to identify ERWTs. First, it is essential to rule out a primary intrarenal tumor with secondary extrarenal metastases or a supernumerary kidney [9–11]. Second, the pathologic assessment should reveal Wilms’ tumor’s characteristic triphasic histologic pattern. Lastly, a comprehensive examination of the entire tumor should demonstrate the absence of teratoma or renal carcinoma [12].**[**Figure 3**]**.

Microscopic analysis typically reveals classical histological features characterized by a triphasic composition encompassing blastema, epithelial, and stromal areas. The blastema, which is the least differentiated component, comprises small, round blue cells with active mitotic activity and overlapping nuclei. This tends to be more prominent in adults and correlates with unfavorable prognoses. The epithelial part shows variable differentiation, ranging from primitive tubular formations to structures resembling nephrogenesis at different stages. The stromal component may consist of densely packed mesenchymal cells or myxoid areas, which exhibit less aggressive behavior [13, 14].

Nephrogenic rests (NR), originating from persistent nephrogenic blastema, are proposed as precursors to Wilms’ tumor [15]. The exact embryological origins of ectopic NRs remain elusive. However, studies indicate that approximately 67% of NRs located in the lumbosacral region are linked with spinal dysraphism [3]. It is hypothesized that these tumors may develop from NRs trapped between the dura and the developing spinal cord, possibly due to neural tube defects impeding renal tissue migration [16]. While the exact pathogenesis remains debatable, the embryonic rest theory emerges as a plausible explanation, elucidating its unusual anatomical location.

To confirm the presence of ERWT, a thorough evaluation of the kidneys using multi-slice spiral CT images is essential to rule out any intrarenal tumors. A double-contrast CT scan is often recommended to ascertain the tumor’s location and assess its resectability. MRI can also be advantageous, particularly for paraspinal and thoracic tumors, when there are symptoms of spinal cord compression [17]. However, relying solely on these imaging modalities cannot provide a definitive diagnosis or accurately differentiate ERWT from other malignancies that may be considered in the differential diagnoses. These may include primary intrarenal tumors with metastasis to extrarenal sites, teratomas containing nephroblastoma elements, and various other primitive mesenchymal tumors, among others [18, 19].

Staging ERWTs is challenging, especially following the National Wilms’ Tumor Study (NWTS) guidelines. These guidelines recommend categorizing all such tumors as stage II or higher due to their location beyond the renal capsule, which necessitates chemotherapy for all patients. While treatment strategies align with those for renal Wilms’ tumors, the unique locations and proximity to adjacent organs introduce distinctive considerations when planning surgical and adjuvant approaches [20]. Managing these complex tumors requires a multidisciplinary approach, integrating diagnostic precision, surgical planning, intraoperative guidance, and postoperative care to address intricacies effectively [20–23].

Due to the limited number of cases, standard management approaches for adults with this condition are lacking. In many specific cases, it is based on pediatric guidelines [24]. Management protocols for this condition typically adhere to guidelines established by the NWTS and the Society of Paediatric Oncology (SIOP) in Europe. The NWTS approach advocates for surgery as the initial step, followed by adjuvant chemotherapy and radiotherapy based on surgical staging. In contrast, SIOP’s approach involves neoadjuvant chemotherapy, followed by surgery and subsequent adjuvant therapies determined by staging. However, in cases of spinal compression, both NWTS and SIOP recommend an initial surgical approach, as in our case. Nevertheless, the effectiveness of these guidelines is still questioned due to the rarity of extrarenal Wilms’ tumors in adults [25, 26].

## Conclusion

In conclusion, this report highlights a unique case of ERWT in an adult, a rare occurrence that challenges conventional assumptions about its typical age of occurrence. It highlights the importance of timely surgical intervention and clinical awareness when encountering unusual masses in similar anatomical locations. Moreover, the correlation between spinal ERWTs and a history of spinal anomalies requires further investigation.

## Data Availability

The data used to support the findings of this case report are available from the corresponding author upon request.
